# Is It Fair to Kill One to Save Five? How Just World Beliefs Shape Sacrificial Moral Decision-making

**DOI:** 10.1177/01461672241287815

**Published:** 2024-10-25

**Authors:** Paul Conway, Rael J. Dawtry, Jason Lam, Ana I. Gheorghiu

**Affiliations:** 1 School of Psychology, University of Southampton, Southampton, UK; 2 Department of Psychology, University of Essex, Colchester, UK; 3 Department of Psychology, University of Portsmouth, Portsmouth, UK

**Keywords:** moral dilemmas, just world beliefs, process dissociation, person perception, morality

## Abstract

Sacrificing a target to save a group violates deontological ethics against harm but upholds utilitarian ethics to maximize outcomes. Although theorists examine many factors that influence dilemma decisions, we examined justice concerns: We manipulated the moral character of sacrificial targets, then measured participants’ dilemma responses and just world beliefs. Across four studies (*N*=1116), participants considering guilty versus innocent targets scored lower on harm-rejection (deontological) responding, but not outcome-maximizing (utilitarian) responding assessed via process dissociation. Just world beliefs (both personal and general) predicted lower utilitarian and somewhat lower deontological responding, but these effects disappeared when accounting for shared variance with psychopathy. Results suggest that dilemma decisions partly reflect the moral status of sacrificial targets and concerns about the fairness implications of sacrificing innocent targets to save innocent groups.

Imagine: As a firefighter at a deadly blaze, you can use your ladder to smash in a floor to save five people, but trap another person. Is this action appropriate? Your answer may depend, in part, on concerns about justice and whether the trapped person “deserves” their fate—for example, by starting the fire. Researchers have identified myriad factors that influence dilemma responses, including perceptions of sacrificial targets (e.g., Cohen & Ann, 2016), and personal beliefs (e.g., Piazza & Landy, 2013). Yet, to our knowledge, no work has examined the role of justice concerns specifically. We did so via two strategies.

First, we manipulated the moral character of sacrificial targets. People may be less willing to sacrifice an innocent person than one responsible for placing the group in jeopardy, due to stronger concern for the suffering of innocent than guilty targets (Gray & Wegner, 2009) and beliefs that guilty targets deserve punishment (Feather, 2006). Conversely, *just world theory* (Lerner, 1980) suggests that people can feel threatened by innocent victims when other courses of action (such as helping them) are unavailable (Haynes & Olson, 2006). Although conceptualized as a coping strategy, just world concerns can lead to devaluation of innocent victims (Sutton et al., 2017). Accordingly, people could paradoxically find it easier to sacrifice an innocent than guilty victim due to the threat they pose. Hence, we measured rejection of sacrificial harm and sensitivity to overall outcomes for guilty versus innocent sacrificial targets.

Second, we examined individual differences in *belief in a just world* (BJW), the tendency to rationalize unfairness by contending that innocent victims deserve to suffer (e.g., Lipkus, 1991). People high in BJW may find sacrificial harm more acceptable via rationalization. Yet it is less clear how BJW may predict concern for group outcomes: BJW may also predict increased concern for groups as part of rationalizing harm to targets. Conversely, as groups are also innocent, people high in BJW may demonstrate reduced concern for both sacrificial targets and beneficiaries. If so, BJW may demonstrate a pattern of dilemma responding similar to dark traits like psychopathy (e.g., Conway et al., 2018). This would be consistent with work showing that BJW predicts harsh social attitudes (e.g., Bizer et al., 2012). We examined whether this possibility holds for different kinds of just world beliefs above and beyond dark traits.

## Sacrificial Dilemmas

Sacrificial dilemmas typically involve causing harm to maximize overall outcomes, such as killing one person to save five. Rejecting harm (i.e., it is not acceptable to trap one person to save five) aligns with *deontological* ethics that determine the morality of an action by its intrinsic nature (Kant, 1959). Accepting sacrificial harm that maximizes outcomes (i.e., trapping one person to save five) is consistent with *utilitarian* ethics that judge the morality of an action by the outcomes it produces (Mill, 1998). Hence, researchers may descriptively refer to dilemma decisions as deontological or utilitarian, insofar as decisions align with relevant philosophical ideals (n.b., the psychological processes that produce dilemma judgments may be very different from those described by philosophers; Conway et al., 2018). Dilemma decisions should not be interpreted as reflections of philosophical values, but rather psychological mechanisms. Whereas classic work focused on a simplistic dual process model, modern research suggests that a wide variety of psychological mechanisms contribute to dilemma decision-making, including subjective evaluations of targets, affective reactions to harm, cognitive evaluations of outcomes, adherence to moral rules, inaction, and self-presentation (e.g., Cohen et al., 2022; Piazza & Landy, 2013; Gawronski, et al., 2013; Rom & Conway, 2018; for a review see Conway, 2023).

Importantly, findings obtained via traditional dilemma research that treats deontological and utilitarian judgments as opposites can remain ambiguous. Instead, we employed process dissociation (PD, Conway & Gawronski, 2013), one of a family of models for describing decision patterns across multiple dilemmas varying key features (see Calanchini et al., 2018). Process dissociation presents two versions of each dilemma manipulating the outcome of sacrificial harm within subjects. This allows for assessing (a) the tendency to reject harm regardless of outcomes (deontology parameter) and (b) sensitivity to differences in outcomes regardless of harm (utilitarian parameter). Importantly, these parameters do not reflect philosophical commitments or psychological mechanisms, but rather patterns of responding that (a) descriptively align with philosophical positions and (b) may themselves reflect a combination of processes.^1^

Process dissociation allows for disentangling whether a given manipulation or predictor selectively influences one response pattern, another, or both (in cases of suppression; for a review see Conway, 2023). For current purposes, we note that manipulations increasing the emotional vividness of sacrificial harm selectively increase harm rejection (deontological) responding (Conway & Gawronski, 2013), suggesting that manipulating characteristics of the sacrificial target should likewise impact only deontological, not utilitarian responding. Furthermore, past work shows that measures related to dark traits, such as psychopathy, egoism, and acceptance of ethics violations, predict lower scores on both parameters, whereas measures of prosocial concern for others, such as moral identity, sensitivity to ethical principles, and rejection of “sin stocks,” predict increased responding on both parameters (Bostyn et al., 2022; Conway et al., 2018; Körner et al., 2020; Reynolds & Conway, 2018; Nizcostca et al., 2022 Reynolds & Conway, 2018, cf. Luke & Gawronski, 2021; Paruzel-Czachura & Farny, 2023).^2^ With such findings in mind, we manipulated target guilt and measured just world beliefs.

## Sacrificial Target Guilt

One variable under-examined in past dilemma research is perceptions of sacrificial targets. Most research examines faceless, genderless strangers (Schein, 2020); exceptions show the importance of personal preferences. For example, Bleske-Rechek and colleagues (2010) found that people are less willing to sacrifice family members, young people, and romantic partners. Uhlmann and colleagues (2009) showed that liberal versus conservative Americans were more willing to sacrifice high-status or American targets to save low-status or non-American targets and vice versa—though more than target considerations drove judgments as many people repeated dilemma judgments when roles reversed. More recently, Cohen and colleagues (e.g., 2016; 2021) measured subjective evaluations of many targets from chimpanzees to one’s mother. They demonstrated that target evaluations are tightly linked to sacrificial decisions and reaction times. Together, these findings suggest that people are highly sensitive to sacrificial target characteristics.

However, existing work does not examine how dilemma decisions shift when harm to the same target results in different outcomes for the beneficiary group; we do so via process dissociation. Moreover, to our knowledge, no research has manipulated the moral character of sacrificial targets. One straightforward prediction is that people will demonstrate stronger aversion to sacrificing innocent rather than guilty targets. This pattern would echo research suggesting that people feel stronger prosocial emotions, such as sympathy and compassion, for innocent victims than guilty perpetrators (e.g., Gray & Wegner, 2009), and that affective aversion to harming targets selectively increases deontological responding (Conway & Gawronski, 2013; Reynolds & Conway, 2019). Theoretically, emotional concern for innocent (versus guilty) targets should increase affect-laden aversion to harming them, thereby selectively increasing deontological responding without impacting utilitarian responding.

However, just world theory also suggests that people are threatened by harm to innocent—but not guilty—victims, which undermines perception that the world is a just place (e.g., Lerner, 1980). Therefore, it remains possible that innocent (versus guilty) victims paradoxically decrease deontological responding, due to emotional distancing. Likewise, people may demonstrate increased utilitarian responding for innocent (versus guilty) targets, along with enhanced evaluations of the beneficiaries of sacrificial action, as a defensive justification for sacrificing innocents. Hence, people may be motivated to view sacrificing innocent victims as “more worthwhile” because the group is worth more, compared to sacrificing guilty victims who do not raise such fairness concerns. We tested these possibilities by manipulating whether sacrificial targets placed the group at risk (Studies 1, 2, 4) or demonstrate negative moral character (Study 3). Furthermore, we measured individual differences in just world beliefs.

## Belief in a Just World

Although people often react to others’ suffering with sympathy and compassion (Haynes & Olson, 2006), people sometimes instead blame or derogate victims (Lerner & Simmons, 1966), especially when their suffering appears severe, prolonged, or uncompensated (for reviews, see Dawtry, et al., 2020; Hafer & Rubel, 2015). Such reactions violate social norms emphasizing compassion toward victims, especially those who suffer due to chance misfortune (Dawtry et al., 2018). This can be understood via *just world theory*, which suggests that people are motivated to believe that the world is fair and people get what they deserve—good (bad) things happen to good (bad) people (Lerner, 1980). Although awareness of innocent suffering can prompt attempts to restore justice (e.g., helping the victim), if the means to do so are unavailable or costly, people may instead rationalize events to maintain a *perception* of justice.

Construing an innocent victim as a “bad” person (derogation), or as having behaved in a way that brought about their suffering (blame), makes their suffering seem deserved and therefore less threatening. Just world theory therefore suggests that, not only do people blame “non-innocent” victims—those who, by virtue of their character or behavior, already seem deserving of suffering—but, to a lesser degree, they also blame innocent victims, to make their suffering appear deserved. Insofar as blaming innocent and non-innocent victims affirms that people are responsible for, and deserving of, the bad outcomes they receive, it serves to affirm and maintain belief in a just world. We accordingly manipulated the guilt or innocence of sacrificial targets to clarify how this manipulation influences dilemma responding.

Whereas some research examines manipulations related to just world theory (e.g., victim innocence), other work examines individual differences in just world beliefs (e.g., Lipkus, 1991; Rubin & Peplau, 1973). People scoring high on such measures have a strong belief that that the world is descriptively fair, and may be especially threatened by, and prone to rationalize, evidence to the contrary. As such, they may demonstrate especially low concern for victims—even innocent victims—to protect this belief, because they readily assume that victims deserve suffering. Just world beliefs may thus predict dilemma responding similar to dark personality traits: reduced concerns for sacrificial victims (low deontology parameter) and reduced concern for group outcomes (low utilitarian parameter, e.g., see Conway et al., 2018; Reynolds & Conway, 2018).

Consistent with this view, general just world beliefs (regarding fairness for others) are often associated with negative social attitudes and behaviors, including victim blame and derogation (e.g., Bizer, Hart, & Jekogian, 2012), authoritarianism (Christopher, et al., 2008; De Keersmaecker & Roets, 2020), vengeance (Ferguson & Kamble, 2012), revenge (Bartholomaeus & Strelan, 2016), vigilantism (Hou, et al., 2017), and harsh punishments (Darley, 2002; Hafer & Gosse, 2010), especially for perpetrators of transgressions (Hafer & Sutton, 2016). Indeed, GBJW motivates or facilitates the domination and manipulation of others (Strelan and Van Prooijen, 2014, Sutton and Winnard, 2007; Sutton et al., 2017), similar to dark traits such as psychopathy (e.g., Levenson et al., 1995). We tested this possibility in Studies 1–3.

That said, just world beliefs may not operate in the same way as dark traits: while psychopathy entails callous disregard for others’ suffering, BJW is conceptualized as a strategy to cope with feeling threatened by others’ suffering, a rationalization that empowers people to pursue long term goals (see Hafer & Rubel, 2015). Therefore, it remains possible that just world beliefs are merely associated with dark traits, yet conceptually distinct. If so, then the association between just world beliefs and dilemma responding may reflect shared variance with dark traits, rather than a direct effect of just world beliefs. We tested this possibility in Study 4.

Furthermore, theorists distinguish between general belief in a just world (GBJW)—the perception that the world is fair for other people—and personal belief in a just world, PBJW—the perception that the world is fair for oneself. Typically, the relationship between GBJW and harsh social attitudes is much stronger than for PBJW, which often correlates with positive attitudes, such as forgiveness, benevolence, concern for victims, helping behavior, long-term goal pursuit, and buffered impacts of tragedy (Bartholomaeus & Strelan, 2016; Callan, et al., 2014; Chobthamkit, et al., 2022). For example, Sutton and Winnard (2007) found that PBJW and GBJW simultaneously predicted lower and higher delinquent intentions, respectively. Hence, PBJW may predict increased deontological and utilitarian responding, in line with other measures of prosocial concern (Conway et al., 2018). We examined these possibilities in Study 4.

## The Current Work

Participants considered moral dilemmas where sacrificial harm would achieve an outcome.

We manipulated the moral character of sacrificial targets between subjects. In Studies 1, 2, and 4 participants learned that the sacrificial target placed the group in danger (guilty condition) or was an innocent bystander (innocent). In Study 3, we described sacrificial targets as performing morally reprehensible (guilty) or laudatory (innocent) actions. We assessed acceptance of sacrificial harm, which we used to compute process dissociation parameters reflecting harm rejection (deontology parameter) and outcome maximization (utilitarian parameter) response tendencies (Conway & Gawronski, 2013).^3^ Finally, we assessed just world beliefs (general BJW: Studies 1–4, personal-BJW & other-BJW, Study 4) to predict dilemma responses and target evaluations. For regressions, we controlled for age and gender (see Friesdorf et al., 2015). Due to concerns over sufficient power to reliably detect interactions with individual difference measures, we interpret such findings with caution (Sommet et al., 2023).^4^

### Data Availability Statement

For each study, we report all manipulations, measures, and exclusions. We preregistered the study design, sample size, and analyses for each study (we also preregistered predictions, but these were not always upheld). All materials, data, and analyses are available on the OSF (https://osf.io/3ajnm/?view_only=357fa15af300497fa8d369e40b55e003).

## Study 1

We manipulated whether sacrificial targets were guilty versus innocent of threatening the group, and measured dilemma decisions, target perceptions, and general BJW. Preregistration: https://aspredicted.org/ny66d.pdf.

### Method

#### Participants

We recruited 215 participants from Prolific Academic, aiming for ~100 per condition. We excluded 14 who failed to complete all dilemmas (see Conway & Gawronski, 2013), seven who failed an attention check (Oppenheimer et al., 2009), and two previously completed the study (total 10 in guilty and 13 in control condition), leaving a final sample of 192 (88 male, 102 female, two non-binary), *M*_age_=33.63 *SD*=10.93. Regarding ancestry, 154 identified as White, 13 as Black, 12 as Asian, 4 as Hispanic, and 9 as Other. A majority (113) hailed from the UK, United States (50), France (2), India (2), and Greece (2). Most reported English was their native language (167) or reported fluency in English (25). According to intxpwr.com (Sommet et al., 2023), *N*=35 would provide 80% power to detect the parameter by condition repeated measures interaction; yet we would need *N*=1025 for 80% power to detect the between-subjects BJW by condition interaction. Therefore, we had sufficient power for main effects and repeated measures but not interaction terms, which we interpret with caution.

#### Procedure and Materials

We employed a 2 (dilemma parameters: deontological vs. utilitarian, within-subjects) × 2 (target: guilty vs. innocent, between-subjects) mixed design (*n*=96 in each between-subjects condition).

##### Moral Dilemmas

Participants read moral dilemmas depicting harmful action to achieve a particular outcome (see OSF for full dilemma text). For each sacrificial action, participants reported either *Yes, this is appropriate* or *No, this is not appropriate*. Participants completed 10 dilemmas in a fixed random order, each with two variants: *incongruent*, where the benefits of harm arguably outweigh the harm caused, and *congruent*, where the benefits do not. For example, the incongruent firefighter dilemma read:

*You are a firefighter and you are the first to respond to a raging apartment fire. The fire has trapped one person on the second floor and five people on the third floor. You can see the building structure weakening; soon it will collapse, killing those inside. All the exits on the third floor are blocked, and the only possible exit on the second floor is a weak spot in the walls. The person on the second floor started the fire for fun. Now they are trying to break out through this weak spot, which will speed up the collapse. In order to save the five people above from dying, you would have to use your fire ladder to smash the walls inward at this spot. Then the five could jump down and out. Unfortunately, if you smash the walls in with your ladder you will trap the person on the second floor, who will die from smoke inhalation. Is it appropriate to use your fire ladder to knock in the walls on the second floor so you can save the five people on the third floor from dying, even though this will kill the person on the second floor?*

The congruent version employed identical wording except that the five people would be saved from injury but not death, leading to the question, *Is it appropriate to use your fire ladder to knock in the walls on the second floor so you can save the five people on the third floor from injury, even though this will kill the person on the second floor?* We tallied how often each participant accepted (versus rejected) sacrificial harm for each variant, then employed the six equations described by Conway and Gawronski (2013) to compute a utilitarian (U) and deontological (D) parameter for each participant (see Appendix for calculation details). The U parameter reflects the tendency to maximize outcomes regardless of causing harm, whereas the D parameter reflects the tendency to consistently reject harm regardless of outcomes.

##### Target Guilt Manipulation

Between subjects, we manipulated whether each sacrificial target was *guilty* or *innocent.* We described guilty targets as knowingly and purposely placing the group at risk; innocent targets had no knowledge or intent to harm others. For example, in the firefighter dilemma, the sacrificial target was described as either responsible for causing the fire or not. Specifically, we added the sentence, *The person on the second floor started the fire for fun.*

##### Target Evaluation

After each dilemma, participants reported feelings of sympathy, compassion, positivity, and favorability towards the sacrificial target on scales from 1 (*Not at all*) to 9 (*Extremely*, α=.99).

##### Just World Beliefs

Next, we assessed perceptions that the world is fair via the seven-item General Belief in a Just World (Dalbert, 1999), e.g., “I feel that people get what they deserve” on scales from 1 (*Strongly disagree*) to 7 (*Strongly agree*, α=.92, randomized order).

##### Demographics

Finally, participants reported age, native language, nationality, ancestry, gender, and previous participation.

## Results

### Dilemma Responding

**Figure 1.**
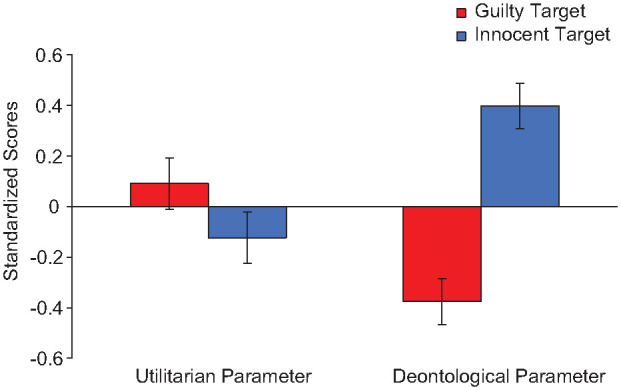
Standardized Utilitarian and Deontological Process Dissociation Parameters for Innocent and Guilty Sacrificial Targets, Study 1. *Note.* Error bars reflect *SE*.

We conducted a 2(Parameter: Utilitarian vs. Deontology) × 2(Condition: Guilty vs. Innocent) mixed ANOVA on the standardized PD parameters, controlling for age and gender (see Figure 1). We found no effect of parameter, *F*(1, 185)=2.21, *p*=.139, η_p_^2^=.01, CI_90%_[.00, .05], but an effect of condition, *F*(1, 185)=8.92, *p*=.003, η_p_^2^=.05, CI_90%_[.01, .10], and interaction, *F*(1, 185)=23.26, *p*<.001, η_p_^2^=.11, CI_90%_[.05, .18].^5^ The utilitarian parameter was not different in the innocent (*M*=−0.13, *SD*=0.82) than guilty condition (*M*=0.10, *SD*=1.10), *F*(1, 185)=2.11, *p*=.148, η_p_^2^=.01, CI_90%_[.00, .05], but the deontology parameter was higher in the innocent (*M*=0.34, *SD*=0.94) than guilty condition (*M*=−0.33, *SD*=0.95), *F*(1, 185)=27.28, *p*<.001, η_p_^2^=.16, CI_90%_[.06, .20].

#### Correlations

A correlational analysis (see Table 1) revealed the typical pattern of PD parameters correlating with conventional utilitarian versus deontological relative judgments but not one another. GBJW correlated negatively with both the deontology and utilitarian parameters, but not target evaluations or age; men scored higher than women. Target sympathy correlated with increased deontological and reduced utilitarian responding, but not age or gender.

**Table 1.** Correlations Between all Measures, Study 1.

Variables	1	2	3	4	5	6	7
1. Relative Utilitarian versus Deontological Judgments	—						
2. Utilitarian PD Parameter	.47***	—					
3. Deontology PD Parameter	–.88***	-.10	—				
4. Belief in a Just World	.16*	–.28***	–.27***	—			
5. Sympathy for Sacrificial Target	–.28**	–.19***	.27***	.07	—		
6. Age	.03	**–**.02	**–**.04	.05	**–**.01	**–**.02	—
7. Gender (1=*f*, 2=*m*)	–.19**	.07	.25***	–.22***	.13	–.17*	**–**.07

*Note.* **p < .05, **p < .01, ***p < .001. PD = process dissociation. Bold values indicates the significance.

#### Regressions

Next, we examined whether GBJW uniquely predicted the harm rejection (deontology) and outcome maximization (utilitarian) parameters across condition, controlling for age, gender, and the other parameter (see Table 2). We entered control variables at step 1, the main effects of GBJW and condition at step 2, and their interaction at step 3. GBJW negatively predicted both parameters; the guilt manipulation reduced only the deontology parameter. We also obtained an unexpected interaction where people higher in GBJW showed lower concern for outcomes (utilitarian parameter) when targets were guilty.^6^

**Table 2.** Regressions Predicting Harm Rejection (Deontology Parameter) and Outcome Maximization (Utilitarian Parameter) Sacrificial Dilemma Response Tendencies from Belief in a Just World and Innocent vs. Guilty Target Condition, and Interaction, Controlling for Age, Gender, and the Other Parameter, Study 1.

	Deontology Parameter					Utilitarian Parameter				
Predictor	β	*t*	*p*	*B* 95% CI LB	*B* 95% CI UB	β	*t*	*p*	*B* 95% CI LB	*B* 95% CI UB
**Step 1**										
Age	–.02	–0.30	.764	–0.02	0.01	.01	0.13	.900	–0.01	0.01
Gender (1 = *male*, 2 = *female*)	**.25**	**3.55**	**<.001**	**0.26**	**0.79**	.12	1.53	.128	–0.07	0.52
Other Parameter	–.11	–1.54	.125	–0.26	0.03	–.12	–1.54	.125	–0.26	0.03
**Step 2**										
Belief in a Just World	**–.23**	**–3.28**	**.001**	**–0.29**	**–0.07**	**–.33**	**–4.55**	**<.001**	**–0.37**	**–0.15**
Condition (0 = *Target Innocent*, 1 = *Guilty*)	**–.35**	**–5.22**	**<.001**	**0.43**	**0.95**	.09	1.14	.257	–0.46	0.13
**Step 3**										
BJW × Condition	.21	0.94	.349	–0.30	0.11	**–.60**	**–2.58**	**.011**	**0.07**	**0.49**

*Note*: Bold indicates significance. For the deontology parameter, the “other parameter” is the utilitarian parameter, and vice versa.

### Discussion

Participants were more willing to sacrifice guilty than innocent targets (i.e., deontological responding), whereas concern for group outcomes remained similar across condition (i.e., utilitarian responding). These findings are consistent with arguments that target preferences play an important role in dilemma decision-making (e.g., Cohen et al., 2022) and furthermore clarify that target guilt does not influence sensitivity to different outcomes of sacrificial harm.

GBJW predicted reduced deontological responding, but GBJW also predicted reduced utilitarian responding—a pattern similar to measures of antisociality (e.g., Conway et al., 2018). We also found an unexpected interaction where GBJW predicted especially low utilitarian responding when sacrificial targets were guilty, suggesting perhaps reduced concern for groups when there is an opportunity to punish guilty targets among people with just world beliefs. However, due to power concerns we interpret such interactions cautiously.

## Study 2

Study 2 replicated Study 1, adding evaluations of both target and beneficiaries. We predicted that people sacrificing guilty versus innocent targets would score lower on the deontology but not utilitarian parameter. We expected GBJW would again predict reduced utilitarian and deontological responding; we remained agnostic regarding interactions.^7^ Preregistration: https://aspredicted.org/i3r6x.pdf.

### Method

#### Participants

We again aimed for >100 per condition. We recruited 249 undergraduates from two UK universities (96 from one; 153 from the other) for partial course credit. We excluded 39 who failed to complete all dilemmas, nine who failed an attention check, and three who previously completed the study (*n*=29 guilty, *n*=22 control), leaving a final sample of 198 (27 male, 164 female, four non-binary, three unreported), *M*_age_=20.24, *SD*=3.80. Regarding ancestry, 140 identified as White, 10 as Black, 28 as Asian, 19 as Other, and one unreported. A majority (142) were from the UK, with the remainder naming countries including India (9), Greece (6), Poland (5), and Italy (4). Most (149) reported English was their native language, with 46 nonetheless reporting fluency in English and two reporting less than fluency.

#### Procedure

We employed the same procedure as Study 1 (*n*=97 guilty, *n*=101 control), except participants completed GBJW (α=.85) before the sacrificial dilemma battery and reported their evaluation of the sacrificial targets and beneficiaries for each dilemma. We asked how positively and favorably participants felt toward each and how much each deserves a positive outcome on scales from 1 (*not at all*) to 7 (*extremely*, α=.99), and the same questions pertaining to each group (α=.98).

### Results

#### Dilemma Responding

**Figure 2.**
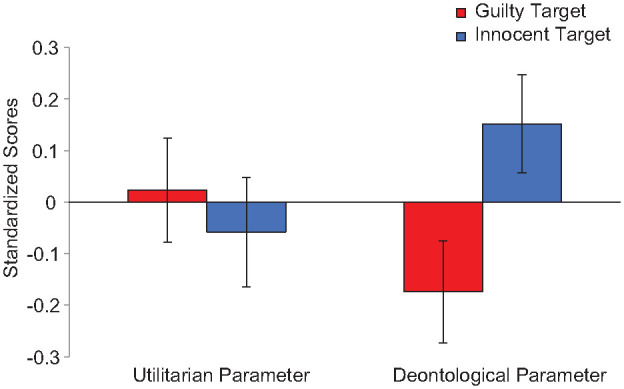
Standardized Utilitarian and Deontological Process Dissociation Parameters for Innocent and Guilty Sacrificial Targets, Study 2. *Note.* Error bars reflect *SE*.

We conducted a 2(Parameter: Utilitarian vs. Deontology, within-subjects) × 2(Condition: Guilty vs. Innocent, between-subjects) mixed ANOVA on the standardized PD parameters controlling for age and gender. We found a theoretically uninteresting main effect of parameter, *F*(1, 186)=6.48, *p*=.012, η_p_^2^=.03, CI_90%_[.01, .09], and no effect of condition, *F*(1, 186)=1.56, *p*=.213, η_p_^2^=.01, CI_90%_[.00, .04], but the interaction was significant, *F*(1, 186)=3.90, *p*=.049, η_p_^2^=.02, CI_90%_[.00, .07] (see Figure 2):^8^ the utilitarian parameter was not different across the innocent (*M*=−0.06, *SD*=0.98) versus guilty condition (*M*=0.02, *SD*=1.03), *F*(1, 186)=0.35, *p*=.556, η_p_^2^< .01, CI_90%_[.00, .03], but the deontology parameter was significantly higher in the innocent (*M*=0.15, *SD*=0.98) than guilty condition (*M*=−0.17, *SD*=0.99), *F*(1, 186)=5.64, *p*=.019, η_p_^2^=.03, CI_90%_[.00, .08].

#### Correlational Analysis

First, we computed correlations between all variables (see Table 3). Again, the PD parameters correlated with relative judgments but not one another. GBJW again correlated negatively with the deontology parameter, though not the utilitarian parameter. The deontology parameter correlated positively with evaluations of targets but negatively with groups; the utilitarian parameter did not correlate with either evaluation. Age (but not gender) correlated with deontological but not utilitarian responding and lower GBJW.

**Table 3.** Correlations Between all Measures, Study 2.

Variables	1	2	3	4	5	6	7
1. Relative Utilitarian versus Deontological Judgments	—						
2. Utilitarian PD Parameter	.51***	—					
3. Deontology PD Parameter	–.86***	–.12	—				
4. Belief in a Just World	.13	–.14	–.25***	—			
5. Evaluation of Sacrificial Target	–.21**	–.06	.18*	.07	—		
6. Evaluation of Group to be Saved	.14	–.02	–.15*	–.09	.12	—	
7. Age	–.29***	–.09	.30***	–.17*	–.01	–.08	—
8. Gender (1=*f*, 2=*m*)	.03	.03	.01	–.04	.13	.15	–.11

*Note.* **p < .05, **p < .01, ***p < .001. PD = process dissociation. Bold values indicates the significance.

#### Regression Analysis

Next, we examined whether GBJW uniquely predicted the harm rejection (deontology) and outcome maximization (utilitarian) parameters across condition, controlling for age, gender, and the other parameter (see Table 4). Replicating Study 1, GBJW negatively predicted both the deontology and utilitarian parameters; however, this time neither interaction was significant. Again, the deontology but not utilitarian parameter was lower for guilty than innocent targets. Younger people also scored higher on the deontology parameter.

**Table 4.** Regressions Predicting Harm Rejection (Deontology Parameter) and Outcome Maximization (Utilitarian Parameter) Sacrificial Dilemma Response Tendencies from Belief in a Just World and Innocent vs. Guilty Target Condition, and Interaction, Controlling for Age, Gender, and the Other Parameter, Study 2.

	Deontology Parameter					Utilitarian Parameter				
Predictor	β	*t*	*p*	*B* 95% CI LB	*B* 95% CI UB	β	*t*	*p*	*B* 95% CI LB	*B* 95% CI UB
**Step 1**										
Age	**.31**	**4.38**	**<.001**	**0.04**	**0.12**	–.05	–0.68	.500	–0.05	0.03
Gender (1 = *male*, 2 = *female*)	.05	0.65	.514	–0.26	0.53	.03	0.37	.711	–0.34	0.49
Other Parameter	–.09	–1.24	.218	–0.22	0.05	–.10	–1.24	.218	–0.25	0.06
**Step 2**										
Belief in a Just World	**–.20**	**–2.82**	**.005**	**–0.32**	**–0.06**	**–.17**	**–2.20**	**.029**	**–0.31**	**–0.02**
Condition (0 = Target *Innocent*, 1 = *Guilty*)	**–.16**	**–2.41**	**.017**	**–0.59**	**–0.06**	.02	0.27	.790	–0.25	0.33
**Step 3**										
BJW x Condition	.23	0.93	.352	–0.14	0.38	.01	0.02	.981	–0.28	0.28

*Note*: Bold indicates significance. For the deontology parameter, the “other parameter” is the utilitarian parameter, and vice versa.

### Discussion

We replicated the finding that sacrificing guilty (versus innocent) targets reduces harm aversion (deontology parameter), without impacting concern for outcomes (utilitarian parameter). Moreover, we replicated the Study 1 finding that GBJW predicted lower scores on both parameters, similar to dark traits (unlike Study 1, GBJW did not interact with condition). However, the fairness violation in these scenarios is egregious: one person places an entire group in mortal jeopardy. Next, we examined whether findings would generalize to a less egregious manipulation. We also measured GBJW before dilemmas and added a measure of how much targets deserve to die.

## Study 3

Studies 1 and 2 showed that people sacrifice guilty targets more than innocent ones, and GBJW predicted reduced concerns for both the target and beneficiary of sacrifice. However, targets were guilty of specifically placing the group in jeopardy; next, we examined whether they would replicate for a more general manipulation of target moral character. The design was identical to Study 1, except all dilemmas were taken from the innocent condition. Instead, we described targets by morally laudable or repugnant behaviors, such as supporting versus stealing from their grandmother. We expected to replicate Study 1, though we were agnostic regarding the interaction between GBJW and condition. Preregistration: https://aspredicted.org/8bz6a.pdf.

### Method

#### Participants

We again aimed for ~100 people per between-subjects condition. We recruited 212 UK participants combined from Prolific for pay (*n*=122) and UK undergraduates for partial course credit (*n*=90). We excluded 30 who failed to complete all dilemmas, seven who failed an attention check, five who previously completed the study, and one with a division by zero error in parameter calculations (23 in bad condition, 20 in good condition), leaving 169 (35 male, 132 female, two non-binary), *M*_age_=28.85, *SD*=12.31. Regarding ancestry, 131 identified as White, 10 as Black, 19 as Asian, and nine as Other ethnicity. Most (120) were from the UK, Poland (9), India (6), and Italy (3). Most (131) reported English was their native language, or (38) fluency in English.

#### Procedure

We measured GBJW before dilemmas. We employed the innocent condition dilemma battery from Study 1, but manipulated between subjects whether sacrificial targets were moral (*n*=84) or immoral (*n*=83). For example, *“This person used to visit his sick grandmother, wait until she fell asleep, then steal cash out of her purse to spend on cigarettes [put some cash in her purse so she could afford her medicine]. He enjoyed the feeling of outsmarting [helping] his grandma and getting things he wanted even though she really needed the money [without making her feel embarrassed for needing money].*” We employed 10 parallel bad and good character descriptions, with each applied to both the congruent and incongruent version of each dilemma (see OSF for full materials). After each dilemma we measured (1=*Not at all* to 9=*Extremely*) participants’ feelings of sympathy and compassion together with target evaluations as positive and favorable (α=.94), and how much the target deserves to die (α=.94).

### Results

#### Dilemma Responding

**Figure 3.**
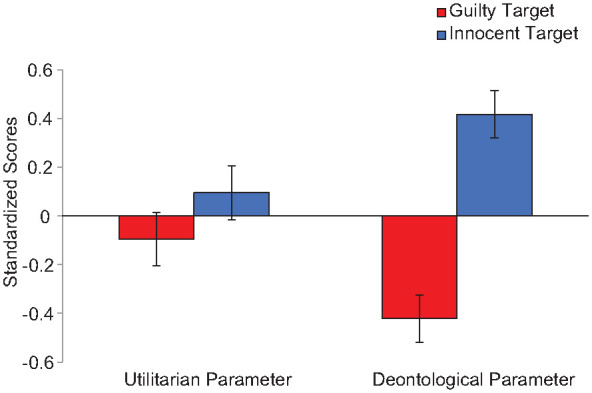
Standardized Utilitarian and Deontological Process Dissociation Parameters for Innocent and Guilty Sacrificial Targets, Study 3. *Note.* Error bars reflect *SE*.

We again conducted a 2(Parameter: Utilitarian vs. Deontology, within-subjects) × 2(Condition: Guilty vs. Innocent, between-subjects) mixed ANOVA on the standardized PD parameters controlling for age and gender. We found a theoretically uninteresting main effect of parameter, *F*(1, 163)=5.29, *p*=.023, η_p_^2^=.03, CI_90%_[.01, .09], a main effect of condition, *F*(1, 163)=23.87, *p*<.001, η_p_^2^=.13, CI_90%_[.06, .21], and significant interaction, *F*(1, 163)=10.34, *p*=.002, η_p_^2^=.06, CI_90%_[.01, .13] (see Figure 3). The utilitarian parameter was not different in the innocent (*M*=−0.09, *SD*=0.98) versus guilty condition (*M*=0.11, *SD*=1.02), *F*(1, 163)=1.54, *p*=.216, η_p_^2^=.01, CI_90%_[.00, .05] but the deontology parameter was higher in the innocent (*M*=0.41, *SD*=0.83) than guilty condition (*M*=−0.43, *SD*=0.98), *F*(1, 163)=37.49, *p*<.001, η_p_^2^=.19, CI_90%_[.10, .27].

#### Correlational Analysis

A correlation analysis (see Table 5) showed the PD parameters again correlated with relative judgments but not one another. GBJW did not correlate significantly with any measure. Target evaluations correlated with deontological but not utilitarian responding; target deservingness correlated negatively with both parameters.

**Table 5.** Correlations Between all Measures, Study 3.

Variables	1	2	3	4	5	6	7
1. Utilitarian versus Deontological Judgments	—						
2. Utilitarian PD Parameter	.44***	—					
3. Deontology PD Parameter	–.80***	.08	—				
4. Belief in a Just World	.06	–.05	–.04	—			
5. Sympathy for Sacrificial Target	–.37***	.10	.46***	–.14	—		
6. Deservingness of Sacrificial Target to Die	.34***	–.18*	–.43***	–.12	–.52***	—	
7. Age	–.17*	.04	.22**	–.02	.07	–.20*	—
8. Gender (1=*f*, 2=*m*)	–.07	–.12	.05	–.11	.03	.04	–.13

*Note.* **p < .05, **p < .01, ***p < .001. PD = process dissociation. Bold values indicates the significance.

#### Regression Analysis

Next, we examined whether GBJW uniquely predicted the harm rejection (deontology) and outcome maximization (utilitarian) parameters across conditions, controlling for age, gender, and the other parameter (see Table 6). Again, the deontology but not utilitarian parameter was lower for guilty than innocent targets. This time, GBJW did not predict either parameter. Nor did we replicate the interaction on the utilitarian parameter; instead, people high in GBJW were less willing to sacrifice guilty than innocent targets. Older people also scored higher on the deontology parameter.

**Table 6.** Regressions Predicting Harm Rejection (Deontology Parameter) and Outcome Maximization (Utilitarian Parameter) Sacrificial Dilemma Response Tendencies from Belief in a Just World and Innocent vs. Guilty Target Condition, and Interaction, Controlling for Age, Gender, and the Other Parameter, Study 3.

	Deontology Parameter					Utilitarian Parameter				
Predictor	β	*t*	*p*	*B* 95% CI LB	*B* 95% CI UB	β	*t*	*p*	*B* 95% CI LB	*B* 95% CI UB
**Step 1**										
Age	**.23**	**2.93**	**.004**	**0.01**	**0.03**	.03	0.36	.723	–0.01	0.02
Gender (1 = *male*, 2 = *female*)	.03	0.35	.728	–0.31	0.44	–.13	–1.69	.093	–0.70	0.05
Other Parameter	.08	1.07	.287	–0.07	0.23	.09	1.07	.287	–0.07	0.24
**Step 2**										
Belief in a Just World	.03	0.36	.721	–0.11	0.16	–.05	–0.62	.533	–0.19	0.10
Condition (0 = *Target Innocent*, 1 = *Guilty*)	**–.42**	**–6.00**	**<.001**	**–1.11**	**–0.56**	–.07	–0.77	.442	–0.48	0.21
**Step 3**										
BJW × Condition	**.56**	**2.18**	**.031**	**0.03**	**0.55**	–.30	–1.01	.313	–0.45	0.15

*Note*: Bold indicates significance. For the deontology parameter, the “other parameter” is the utilitarian parameter, and vice versa.

### Discussion

Study 3 replicated the finding that people were more willing to sacrifice guilty than innocent targets (lower deontological responding), whereas concern for groups remained similar (utilitarian responding) even for less egregious moral violations. However, inconsistent with Studies 1 and 2, GBJW did not predict either parameter, and the interaction now showed people high in GBJW were more willing to sacrifice innocent than guilty targets (low deontology parameter). Between inconsistent interactions and power concerns we interpret this pattern cautiously.

One possibility why effects did not replicate is that the less egregious moral character manipulation influenced how just world threat is best served (e.g., by denigrating the innocent rather than punishing the guilty). Completing GBJW immediately before dilemmas or asking about deservingness of death influenced responding may also have influenced self-presentation concerns (Rom & Conway, 2018). Either way, Study 4 returned to the Study 1 manipulation, though evaluating both target and beneficiary.

## Study 4

Studies 1 and 2 (albeit not 3) showed that just world beliefs predicted reduced deontological and utilitarian responding, similar to dark traits. One explanation for this pattern is that BJW operates like a dark trait—reflecting a cynical, callous disregard for others (Moshagen, et al., 2018). If so, just world beliefs should predict above and beyond measures of dark traits. Alternately, BJW may primarily reflect a coping strategy for managing uncertainty and that merely covaries with dark traits in some cases and may elsewise diverge (Haynes & Olson, 2006). If so, including dark traits in a regression should eliminate the predictive power of just world beliefs. Moreover, a host of research suggests that links to antisociality are limited to general belief in a just world (GBJW), belief the world is fair for others; personal belief in a just world (PBJW) appears linked to prosocial concern (e.g., Sutton et al., 2017). Therefore, we anticipated that GBJW and PBJW may demonstrate opposite predictive patterns. We tested these possibilities in Study 4.

We again measured just world beliefs in general (GBJW), but we added measures of just world beliefs for oneself (personal, PBJW) and for others (others, OBJW), as well as psychopathy (Levenson, et al., 1995). We predicted that people sacrificing guilty versus innocent targets would again demonstrate lower concern about rejecting sacrifice (deontological parameter) but not maximizing outcomes (utilitarian parameter). We predicted that GBJW, OBJW, and psychopathy would predict lower D scores (main effects), and possibly lower U scores (main effect), whereas PBJW will predict both higher D and possibly U scores (main effects), in line with measures of moral concern (e.g., see Conway, 2018). To maximize sample size on a modest budget and increase generalizability, we recruited from the largest English-speaking country on earth: India. BJW findings often replicate across culture and context (Bartholomaeus et al., 2023; Bollmann et al., 2015; Chobthamkit, et al., 2022), as do sacrificial decision-making findings (Awad et al., 2020). Hence, we anticipated replication despite potential cultural differences between UK and Indian samples (though culture may play a role; see Discussion). Preregistration: https://aspredicted.org/v4ye3.pdf.

### Method

#### Participants

To increase power, we aimed for 300 participants per condition, i.e., 600 participants. We recruited English-speaking Indian participants via Besample (www.besample.app), for $0.50 USD. Although 996 participants began the study, fewer completed it. As preregistered, we excluded 300 who failed to complete all dilemmas (who averaged 47% complete), 131 who failed an attention check, and eight with a division-by-zero error in parameter calculation, leaving 557 (383 male, 171 female, three unspecified other gender), *M*_age_=30.55, *SD*=9.48.^9^ Regarding ancestry, 531 identified as Indian or from the Asian subcontinent, 14 as White, and 12 as other ethnicities, “human,” or unreported. A majority (545) reported living in India, six in other countries, and six unreported, with 45 native English speakers, 471 fluent non-native speakers, and 40 less than fluent (one unreported).

#### Procedure and Materials

Participants again considered congruent and incongruent dilemmas where sacrificial targets were either guilty (*n*=272) or not (*n*=285) of placing others at risk. Participants indicated whether sacrificial action was acceptable and evaluated targets (α=.98) and groups (α=.98) as in Study 2. Participants again completed the Dalbert GBJW measure (α=.82), plus eight Personal Belief in a Just World (PBJW) items (α=.88, e.g., *I feel that the world treats me fairly*) and eight Other Belief in a Just World (OBJW) items (α=.90, *I feel that the world treats people fairly*, in a random order on scales from 1=*Strongly disagree* to 7=*Strongly agree* (Lipkus et al., 1996). Participants also completed 16 items measuring psychopathy on the same scale (α=.79), such as *Success is based on survival of the fittest; I am not concerned about the losers* (Levenson et al., 1995).

### Results

#### Dilemma Responding

**Figure 4.**
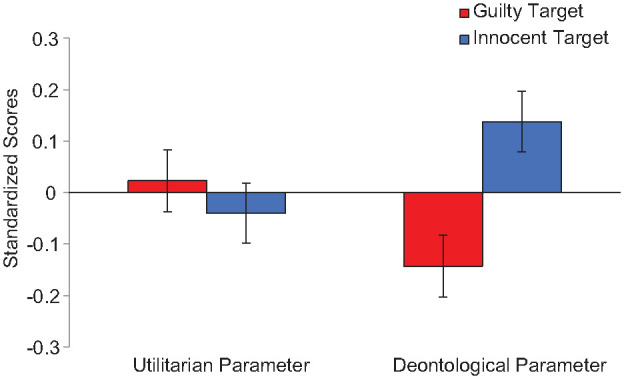
Standardized Utilitarian and Deontological Process Dissociation Parameters for Innocent and Guilty Targets, Study 4. *Note.* Error bars reflect *SE*.

We conducted a 2(Parameter: Utilitarian vs. Deontology, within-subjects) × 2(Condition: Guilty vs. Innocent, between-subjects) mixed ANOVA on the standardized PD parameters controlling for age and gender. We found no main effect of parameter, *F*(1, 549)=0.12, *p*=.714, η_p_^2^<.01, CI_90%_[.00, .01], or condition, *F*(1, 549)=3.16, *p*=.076, η_p_^2^=.01, CI_90%_[.00, .02], but we found the expected interaction, *F*(1, 549)=9.09, *p*=.003, η_p_^2^=.02, CI_90%_[.00, .04] (see Figure 4).^10^ As predicted, the deontology parameter was significantly higher for innocent (*M*=.14, *SD*=0.99) than guilty sacrificial targets (*M*=−0.14, *SD*=0.98), *F*(1, 549)=11.12, *p<*.001, η_p_^2^=.02, CI_90%_[.01, .04], whereas the utilitarian parameter was not (Innocent: *M*=−0.04, *SD*=0.99; guilty: *M*=0.02, *SD*=.97), *F*(1, 549)=0.56, *p*=.456, η_p_^2^<.01, CI_90%_[.00, .01].

#### Correlational Analysis

Next, we computed correlations between all variables (see Table 7). Again, the PD parameters correlated with relative judgments but not one another, and patterns that emerged on the parameters combined to show similar patterns on relative judgments. All three BJW measures correlated highly with one another, and moderately with psychopathy. GBJW again correlated negatively with both the deontological and utilitarian parameters, as did OBJW. Conversely, PBJW correlated negatively with the utilitarian but not deontological parameter. Psychopathy also correlated negatively with both parameters. The deontology parameter again correlated positively with evaluations of targets but negatively with groups; the utilitarian parameter again did not correlate with either. Age and gender were uncorrelated with dilemma responding, older individuals scored higher on OBJW and PBJW.

**Table 7.** Correlations Between all Measures, Study 4.

Variables	1	2	3	4	5	6	7	8	9	10
1. Relative Utilitarian vs. Deontological Judgments	—									
2. Utilitarian PD Parameter	.15***	—								
3. Deontology PD Parameter	–.96***	.07	—							
4. General BJW	.06	–.16***	–.08*	—						
5. Personal BJW	.05	–.11*	–.07	.72***	—					
6. Other BJW	.09*	–.13**	–.12**	.80***	.82***	—				
7. Psychopathy	.18***	–.19***	–.22***	.52***	.43***	.55***	—			
8. Evaluation of Sacrificial Target	–.16***	**–**.13**	.12*	.15**	.13***	.17***	.12**	—		
9. Evaluation of Group to be Saved	–.01	–.02	–.01	–.02	.02	.01	–.02	.54***	—	
10. Age	.03	.04	–.03	.07	.09*	.09*	.01	.09*	.10*	—
11. Gender (1=*f*, 2=*m*)	–.02	–.03	.02	.00	–.07	–.05	.01	.06	.10*	.06

*Note.* **p < .05, **p < .01, ***p < .001. BJW = belief in a just world, PD = process dissociation.

#### Regression Analysis

As preregistered, we individually regressed the D and U parameters on GBJW, PBJW, OBJW, psychopathy, and condition (at step 2), plus the interaction between condition and each predictor (step 3), controlling for age, gender, and the other parameter (step 1). Individually, GBJW predicted significantly lower scores on the utilitarian parameter, β =−.17, *t*=−4.17, *p*<.001, but not deontological parameter, β =−.08, *t*=−1.89, *p*=.060. Likewise, PBJW predicted significantly lower utilitarian, β =−.10, *t*=−2.41, *p*=.016, but not deontological scores, β =−.06, *t*=−1.42, *p*=.157. Conversely, OBJW predicted both significantly lower utilitarian, β =−.15, *t*=−3.60, *p*<.001, and deontology parameter scores, β =−.12, *t*=−2.89, *p*=.004. Psychopathy predicted both significantly lower utilitarian, β =−.19, *t*=−4.48, *p*<.001, and deontology parameter scores, β =−.21, *t*=−5.13, *p*<.001. These findings generally align with preregistered hypotheses of BJW acting similar to psychopathy in predicting lower scores on one or both parameters—although preregistrations anticipated stronger effects on the deontological parameter, whereas results showed stronger effects on the utilitarian parameter.

However, we also preregistered regressing each parameter on all predictors simultaneously (see Table 8). In this analysis, no BJW variable significantly predicted either parameter—only psychopathy remained a significant predictor of lower deontological and lower utilitarian responding. Although we did not preregister interaction predictions due to power concerns, we nonetheless replicated the Study 1 interaction that people high in GBJW showed lower concern for outcomes (utilitarian parameter) when targets were guilty versus innocent. In addition, people high in psychopathy showed higher concern for outcomes (utilitarian parameter) when targets were guilty versus innocent. Nonetheless, we caution against interpretation considering power.

**Table 8.** Regressions Predicting Harm Rejection (Deontology Parameter) and Outcome Maximization (Utilitarian Parameter) Sacrificial Dilemma Response Tendencies from Belief in a Just World (General, Personal, and Other), Psychopathy, and Innocent vs. Guilty Target Condition, and their Interactions, Controlling for Age, Gender, and the Other Parameter, Study 4.

	Deontology Parameter					Utilitarian Parameter				
Predictor	β	*t*	*p*	*B* 95% CI LB	*B* 95% CI UB	β	*t*	*p*	*B* 95% CI LB	*B* 95% CI UB
**Step 1**										
Age	–.03	–0.79	.433	–0.00	0.00	.05	1.24	.215	–0.00	0.00
Gender (1 = *male*, 2 = *female*)	.03	0.58	.562	–0.05	0.08	–.03	–0.61	.540	–0.04	0.02
Other Parameter	.07	1.72	.086	–0.00	0.06	.07	1.72	.086	–0.00	0.03
**Step 2**										
General BJW	.07	0.96	.336	–0.02	0.07	–.14 ^a^	–1.92	.055	–0.05	0.00
Personal BJW	.09	1.22	.222	–0.02	0.07	.09 ^a^	1.23	.218	–0.01	0.04
Other BJW	–.14 ^a^	–1.55	.121	–0.09	0.01	–.05 ^a^	–0.52	.602	–0.04	0.02
Psychopathy	**–.21** ^a^	**–4.24**	**< .001**	**–0.15**	**–0.06**	**–.13** ^a^	**–2.59**	**.010**	**–0.06**	**–0.01**
Condition (0 = *Innocent*, 1 = *Guilty*)	**–.15** ^a^	**–3.60**	**< .001**	**–0.17**	**–0.05**	.03	0.65	.514	–0.02	0.04
**Step 3**										
GBJW × Condition	.32	1.01	.314	–0.04	0.14	**–.62**	**–1.98**	**.049**	**–0.09**	**–0.00**
PBJW × Condition	–.45	–1.43	.153	–0.16	0.03	.46	1.46	.144	–0.01	0.09
OBJW × Condition	.31	0.88	.379	–0.06	0.15	–.49	–1.39	.166	–0.09	0.02
Psychopathy × Condition (0, 1)	–.35	–1.13	.259	–0.15	0.04	**.99**	**3.25**	**.001**	**0.03**	**0.13**

*Note*: Bold indicates significance. BJW = belief in a just world. For the deontology parameter, the “other parameter” is the utilitarian parameter, and vice versa. ^a^ denotes effects that are significant (negative) predictors in individual regressions.

### Discussion

Study 4 replicated and clarified Studies 1–3 in a larger sample from a different country. We again found that people were more willing to sacrifice guilty than innocent targets (lower deontology parameter), without changes to group outcomes (utilitarian parameter). Study 4 also replicated but clarified the role of just world beliefs. In individual regressions, each of the three just world predictors, GBJW, PBJW, and OBJW negatively predicted the utilitarian parameter (OBJW additionally negatively predicted the deontology parameter)—however, in a multiple regression together with psychopathy, none of these effects remained significant. Instead, psychopathy negatively predicted both the deontology and utilitarian parameters, consistent with past work (e.g., Conway et al., 2018). This pattern suggests that Study 1 and 3 findings (GBJW predicted lower dilemma responding) were due to shared variance with dark traits—rather than just world beliefs directly predicting responses. Just world beliefs may not operate like a dark trait, but rather correlate (substantially) with dark traits that impact dilemma responses in predictable ways. In other words, this pattern may be due to people high in dark traits tending to endorse BJW, rather than BJW itself operating as a dark trait.

Finally, we obtained two unexpected interactions: replicating Study 1, GBJW predicted especially low utilitarian responding when sacrificial targets were guilty, whereas psychopathy predicted especially high utilitarian responding when sacrificial targets were guilty. Power concerns require cautious interpretation. However, they tentatively suggest a dissociation between just world beliefs and dark traits.

## General Discussion

Across four studies, we found that people were more willing to sacrifice guilty than innocent targets (i.e., lower deontological responding), but target guilt did not impact sensitivity to maximizing outcomes (utilitarian responding). This pattern held across multiple moral character manipulations and samples from the UK and India, consistent with work showing that BJW findings (Bartholomaeus et al., 2023; Bollmann et al., 2015; Chobthamkit, et al., 2022) and sacrificial dilemma findings (Awad et al., 2020) emerge cross-culturally. Moreover, we found that individual differences in just world beliefs typically (but not always) predicted a pattern of lower deontological and utilitarian responding, similar to dark traits like psychopathy (Conway et al., 2018). This pattern held for general belief in a just world (GBJW) and other belief in a just world (OBJW), measures of how fair people believe the world is for others, as well as personal belief in a just world (PBJW), belief the world is fair for oneself. This pattern emerged across Studies 1 and 2 and largely held in Study 4—but including psychopathy in the regression model rendered all just world belief measures non-significant. This pattern suggests that just world beliefs predict dilemma responding due to shared variance with dark traits, i.e., people high in dark traits endorse just world beliefs, but not everyone high in just world beliefs acts consistent with dark traits.

### Implications

These findings have several implications. First, insofar as aversion to causing harm is one among many factors influencing dilemma judgments (Reynolds & Conway, 2018), these findings align with classic arguments that people feel stronger concern for harm to innocent than guilty victims (Gray & Wegner, 2009) and feel guilty targets deserve to suffer (Feather, 2006). We did not find evidence of a paradoxical reduction in concern for sacrificing innocent (versus guilty) victims that could result from the threat to just world beliefs that innocent victims pose (Lerner & Simmons, 1966). These findings respond to a call for dilemma studies to consider the importance of social roles and relationships (Schein, 2020) and are broadly in line with work showing that target evaluations are important in moral decision-making, as people often sacrifice or save targets they personally prefer (e.g., Cohen et al., 2022). However, the current findings go beyond past work by showing that target evaluations can emerge from the relationship between target and group (e.g., the target started a fire that placed the group at risk) rather than evaluations of the targets in isolation. Moreover, the current work goes beyond past work by employing modeling to examine how sacrificial decisions regarding the same target vary depending on different outcomes of sacrifice.

Nor did we find evidence that people higher in just world beliefs elevated evaluation of the beneficiaries of sacrificial action to increase justification of sacrificial harm. Conversely, people high in just world beliefs appeared less concerned with either sacrificing individual targets or with benefitting the group—consistent with arguments that just world beliefs can promote harsh social attitudes including derogation of victims (note that in dilemmas both targets and beneficiaries face victimization (e.g., Bizer, et al., 2012; Christopher, et al., 2008; Bartholomaeus & Strelan, 2016; Darley, 2002; Hafer & Sutton, 2016; Strelan & Van Prooijen, 2014, Sutton & Winnard, 2007; Sutton et al., 2017). This pattern parallels the pattern demonstrated by dark traits and antisocial thinking, such as psychopathy, egoism, and acceptance of ethical transgressions (e.g., Bostyn et al., 2022; Conway et al., 2018; Gawronski, et al., 2017; Körner et al., 2020; Nizcostca et al., 2022; Reynolds & Conway, 2018).

The impact of just world beliefs on dilemma responding might suggest they operate as a dark trait. However, just world beliefs are conceptualized as a coping strategy for managing the uncertainty of long-term investment when hard work and effort appear unrewarded in one’s environment (e.g., Hafer & Rubel, 2015). People high in just world beliefs may cope with such uncertainty by devaluing both the targets and beneficiaries in sacrificial dilemmas, to manage the threat posed by sacrificial harm. Notably, this pattern parallels the pattern of people who report high childhood social unpredictability (Maranges et al., 2021). Childhood unpredictability tends to motivate reduced concern for others and long-term planning in favor of maximizing immediate personal gains as a coping strategy for uncertainty. Furthermore, people high in anxious or avoidant attachment styles, likewise conceptualized as coping strategies for managing particular interpersonal relationships, also show the same low-deontology, low-utilitarian pattern (Maranges et al., 2022).

Moreover, in Study 4, when including psychopathy in the regression model, all measures of just world beliefs became non-significant, suggesting that the impact of just world beliefs on dilemma responding may be due to shared variance with dark traits (which they correlated moderately with), rather than a direct impact themselves. People high in psychopathy may tend to endorse just world beliefs, which can allow for dismissal of suffering consistent with dark trait thinking. But that is not to say that everyone high in just world beliefs endorses dark trait thinking—many may rely on BJW for coping as originally theorized. Consistent with this view, Armstrong (2019) found that just world beliefs only predicted disregard for victim suffering when participants were ambivalent about how fair the world prescriptively “should” be (in line with dark traits). Other participants high in both descriptive and prescriptive just world beliefs remained sensitive to suffering, suggesting motivated concerns about justice in line with classic theory operate differently than dark traits (Lerner, 1980). That said, the correlation between just world beliefs and dark traits was stronger than correlations in other samples (e.g., Bizer et al., 2012). It is possible this relationship is stronger in Indian than American samples; future work should extend generalizability and clarify how robust this interpretation is.

Although the impact of just world beliefs on dilemma responding held across both Studies 1, 2, and 4, where the target was directly responsible for placing the group at risk, they did not emerge in Study 3, where the target was not responsible for threatening the group. It is unclear why findings did not emerge in this study. It could be that the less egregious manipulation impacted just world concerns. It could also be that the experience of completing dilemmas itself activates just world concerns, increasing the predictive power of the measure, so by measuring BJW first in this study we limited the ability to predict. Future work may clarify boundary conditions on the predictive power of just world beliefs for dilemma judgments.

Surprisingly, the predictive pattern of general belief in a just world (GBJW) largely extended to personal belief in a just world (PBJW), which is typically associated with more positive and prosocial considerations (e.g., Strelan & Sutton, 2011; Bartholomaeus & Strelan, 2019). Although we predicted that PBJW would likewise show a pattern of dilemma responding associated with prosocial concern for others—high deontological and utilitarian responding (Conway et al., 2018)—PBJW instead predicted lower utilitarian responding, similar to GBJW and belief in a just world for others (OBJW), until psychopathy was included in the model. Moreover, all three just world measures correlated substantially with psychopathy. These findings raise questions about whether PBJW always entails increased prosocial considerations.

The interaction between just world beliefs and target guilt were not consistent across studies. The only somewhat consistent finding was that in Study 1 and 4, GBJW predicted especially low utilitarian responding when sacrificial targets were guilty. In Study 4, psychopathy showed the opposite interaction: psychopathy predicted especially high utilitarian responding when sacrificial targets were guilty. Due to power concerns and inconsistency—GBJW did not show this pattern in studies 2 and 3—we interpret such interactions cautiously. Although Study 4 had the largest sample, it still provides less than 80% power to detect an interaction of this structure (Sommet et al., 2023). However, this pattern tentatively suggests a dissociation between just world beliefs and dark traits, with just world beliefs tied to reduced focus on the group when targets are guilty—suggesting target guilt may be sufficient motivation for people high in just world beliefs, whereas psychopathy entails increased focus on groups when victims are guilty, perhaps suggesting increased justification for harming guilty targets. However, such inferences remain speculative.

### Limitations

Theorists have criticized dilemmas for hypotheticality and lacking ecological validity (e.g., Schein, 2019). Nonetheless, dilemmas remain useful as artificial stimuli for probing moral thinking, much like artificial visual stimuli (e.g., red triangles) remain useful for probing the visual system (Cushman & Greene, 2012). Moreover, sacrificial dilemmas align with psychological experiences in real world cases where causing harm will maximize overall outcomes, including military operations, healthcare decisions, and some government policies (Conway, 2023). Therefore, it remains interesting and useful to clarify how perceptions of victims and just world beliefs influence dilemma decisions.

Another limitation is that the current work employs process dissociation (PD), which is related to a growing family of more complex models that estimate additional parameters. For example, the consequences, norms, and generalized inaction (CNI) model adds dilemmas where refusing to help a target maximizes outcomes (or not), allowing for estimating sensitivity to norms (i.e., consistently favoring the focal target), sensitivity to consequences (i.e., consistently maximizing outcomes), and general inaction tendencies (rejecting all action regardless of target and outcome, Gawronski et al., 2017). In this model, psychopathy reliably predicts reduced sensitivity to norms, sometimes reduced sensitivity to consequences, and generally reduced inaction (Körner et al., 2020; Luke & Gawronski, 2021), though some studies find more complex patterns (Luke et al., 2022; Paruzel-Czachura & Farny, 2023). If the current findings replicate using the CNI model, just world beliefs may predict a lower norms parameter, lower consequences parameter, and possibly lower inaction parameter. Future work may clarify such patterns by using GBJW to predict responses using the CNI model.

Third, the current work differs from conventional just world belief work in an important sense: Typically, BJW participants react to suffering where the victim’s fate has already been determined, allowing victim derogation to restore a sense of justice (when other means are unavailable, Haynes & Olson, 2006). Conversely, in the dilemma literature, people decide what should happen—the fate of sacrificial targets and beneficiaries has not yet been determined. The request to decide whether someone should suffer may conflict with just world concerns—if people deserve what they get, then presumably choosing to avoid involving oneself may be most in line with defending one’s own worldview. That said, this perspective suggests that GBJW should predict increased deontological responding as people high in GBJW refuse to involve themselves in sacrificial harm—the opposite of what the current data suggest. Therefore, although the current study examines prospective rather than retrospective harm, current results cannot be explained by people high in GBJW refusing to cause injustice.

## Conclusions

The current work tested how just world beliefs predicted decisions in sacrificial moral dilemmas where causing harm does or does not maximize outcomes, and sacrificial targets vary in terms of guilt or innocence. As expected, people were less willing to sacrifice innocent than guilty targets, a pattern that exclusively loaded on harm rejection (deontological) response tendencies without impacting outcome-maximization (utilitarian) tendencies. Although the predictive impact of just world beliefs was inconsistent, when it was significant it was negatively associated with both deontological and utilitarian responding, a pattern similar to measures of antisociality such as psychopathy and egoism. Therefore, the current findings contribute to a body of work suggesting that just world beliefs impact dilemma responding via association with dark traits.

## Figures and Tables

**Figure A1.**
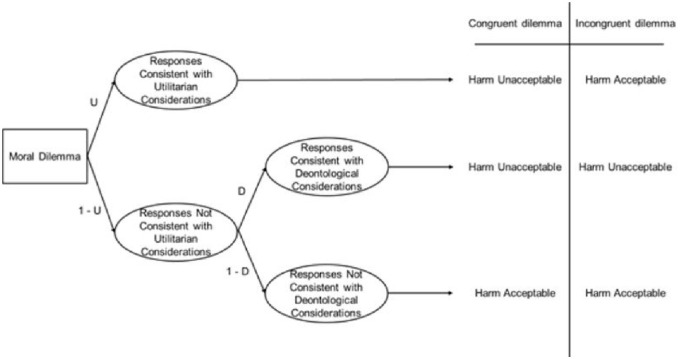
Processing Tree Illustrating the Elements Underlying Responses To Congruent And Incongruent Sacrificial Dilemmas.

## Appendix: Process Dissociation Calculations

Calculating the deontology and utilitarian PD parameters entails recording harm acceptance and rejection responses for congruent and incongruent dilemmas. Harmful action maximizes outcomes for incongruent, but not congruent, dilemmas. Thus, responding consistent with utilitarianism entails accepting harm on incongruent dilemmas but rejecting harm on congruent dilemmas; responding consistent with deontology entails consistently rejecting harm.

In the processing tree illustrated in Figure A1, the top path illustrates responses consistent with utilitarianism: rejecting harm for congruent dilemmas but accepting harm for incongruent dilemmas. The second path illustrates responses consistent with deontology: rejecting harm for both congruent and incongruent dilemmas. The bottom path represents responses consistent with neither utilitarianism nor deontology, thus allowing indiscriminate harm to occur.

The columns on the right allow for formally describing the cases that can lead people to accept or reject sacrificial harm. For congruent dilemmas, people reject harm when responses are consistent with either utilitarianism, *U*, or deontology, *(1 – U) × D*; people accept harm only when responses are consistent with neither, *(1 – U) × (1 – D)*. For incongruent dilemmas, people reject harm when responses are consistent with deontology, *(1 – U) × D*, but accept harm when consistent with either utilitarianism, *U*, or neither utilitarianism nor deontology, *(1 – U) × (1 – D)*. These patterns can be described via four equations:

The probability of rejecting harm on congruent dilemmas is represented as: p(unacceptable|congruent)=U+[(1−U)×D]
The probability of accepting harm on congruent dilemmas is represented as: p(acceptable|congruent)=(1−U)×(1−D)
The probability of rejecting harm on incongruent dilemmas is represented as: p(unacceptable|incongruent)=(1−U)×D
The probability of accepting harm on incongruent dilemmas is represented as: p(acceptable|incongruent)=U+[(1−U)×(1−D)]

Rearranging these four equations allows for algebraically solving for the two unknown variables, *U* and *D*, as follows:

$$\begin{array}{l} U=p\;(unacceptable\;\;|\;\;congruent)\;\\ \;\;\;\;\;\;\;\;-\;p\;(unacceptable\;\;|\;\;incongruent) \end{array}$$

Then plugging in this value of *U* allows for computing *D*:

D=p(unacceptable|incongruent)/(1−U)

Together, these six equations allow for converting acceptability responses to congruent and incongruent dilemmas into independent estimates of the degree to which responses align with utilitarian concerns (maximizing outcomes regardless of causing harm) and deontological concerns (rejecting harm regardless of outcomes).

## Notes

1. We retain this suboptimal naming convention to maintain consistency with past work.

2. Critics argue that some participants disagree whether some sacrifices qualify as “worthy” from a utilitarian perspective, noting so-called “perverse responding,” i.e., accepting sacrificial harm on congruent dilemmas with less positive outcomes (Baron & Goodwin, 2020). However, modeling approaches simply describe response patterns. It is not required that participants and researchers agree, only that responses to dilemmas systematically relate to psychological constructs (Gawronski et al., 2020); e.g., people high in psychopathy tend to accept “perverse responding,” whereas people high in moral identity reject it (Conway et al., 2018).

3. We also measured evaluations of sacrificial targets (Studies 1–4), how much targets deserve to die (Study 3), and evaluations of sacrificial beneficiaries, i.e., the group to be saved (Studies 2 and 4). For space considerations we present these findings in a supplement.

4. We originally preregistered BJW by condition interactions, which were rarely supported, and should be interpreted cautiously due to power.

5. We present 90% CIs as recommended by Steiger (2004).

6. Preregistered mediation via sympathy was not significant so will not be discussed further.

7. We ran Study 3 first and preregistered replicating the interaction but lacked power to adequately test this claim.

8. The interaction remains significant without controls, *p*=.046, and including attention check failures, *p*=.031.

9. We unfortunately did not record recaptcha. No participant not already excluded had zero variation.

10. The interaction remains significant without controls, *p*=.002, and including attention check failures, *p*=.002.
